# Evaluation Of A Positive Psychoeducational Intervention For Informal Caregivers: Randomized Controlled Trial

**DOI:** 10.1093/geroni/igaf122.3929

**Published:** 2025-12-31

**Authors:** Yoko Moriyama, Akemi Matsuzawa, Tomoko Wakui, Nanako Tamiya, Hideto Takahashi

**Affiliations:** National Institute of Public Health, Wako, Saitama, Saitama, Japan; Hokkaido University, Sapporo, Hokkaido, Japan; Tokyo Metropolitan Institute for Geriatrics and Gerontology, Tokyo, Tokyo, Japan; University of Tsukuba, Tsukuba, Ibaraki, Japan; Teikyo Heisei University, Nakano, Tokyo, Japan

## Abstract

The purpose of this study is to evaluate the impact of a positive psychology-based online program on the well-being of working-age informal caregivers. We have developed a positive psychoeducational program to enhance the well-being of working-age informal caregivers because we have confirmed it is low. We incorporated three themes in positive psychology—strengths, self-compassion, and best possible selves into the program and, set a two-month implementation period. Five hundred caregivers of older family aged 30 to 59, recruited from a web-based research company, were randomly assigned to an intervention group of 350 and a control group of 150. Surveys on well-being were conducted at four points: baseline, mid-program, post-program, and one month later: follow-up. The dependent variable was the change in Subjective Happiness Scale: SHS scores from baseline to 1) post-program and 2) follow-up, with program participation as the independent variable. Multiple regression analysis was performed, adjusting for baseline SHS, caregiver age, cohabitation status, older family age, care level, dementia, primary caregiver or not, and caregiving frequency. One hundred eighty intervention participants worked on all sessions and 118 control participants responded to the end point survey, and 167 and 104 of whom responded to the follow up survey. At the end point, a significant positive correlation was observed among change in SHS and intervention (coefficient;0.187, P-value;0.036), however at the follow up, it was not observed (-0.04, 0.717). The effect of intervention was observed immediately after implementation, but it was not sustained, indicating further efforts are needed to maintain the effects.

